# Can Unmanned Aerial Systems (Drones) Be Used for the Routine Transport of Chemistry, Hematology, and Coagulation Laboratory Specimens?

**DOI:** 10.1371/journal.pone.0134020

**Published:** 2015-07-29

**Authors:** Timothy K. Amukele, Lori J. Sokoll, Daniel Pepper, Dana P. Howard, Jeff Street

**Affiliations:** 1 Department of Pathology, Johns Hopkins University School of Medicine, Baltimore, Maryland, United States of America; 2 Clinical Core Laboratory at Infectious Diseases Institute, Makerere University-Johns Hopkins University, Kampala, Uganda; 3 University of Michigan Medical School, Ann Arbor, Michigan, United States of America; 4 NextGen Aeronautics, Torrance, California, United States of America; Gentofte University Hospital, DENMARK

## Abstract

**Background:**

Unmanned Aerial Systems (UAS or drones) could potentially be used for the routine transport of small goods such as diagnostic clinical laboratory specimens. To the best of our knowledge, there is no published study of the impact of UAS transportation on laboratory tests.

**Methods:**

Three paired samples were obtained from each one of 56 adult volunteers in a single phlebotomy event (336 samples total): two tubes each for chemistry, hematology, and coagulation testing respectively. 168 samples were driven to the flight field and held stationary. The other 168 samples were flown in the UAS for a range of times, from 6 to 38 minutes. After the flight, 33 of the most common chemistry, hematology, and coagulation tests were performed. Statistical methods as well as performance criteria from four distinct clinical, academic, and regulatory bodies were used to evaluate the results.

**Results:**

Results from flown and stationary sample pairs were similar for all 33 analytes. Bias and intercepts were <10% and <13% respectively for all analytes. Bland-Altman comparisons showed a mean difference of 3.2% for Glucose and <1% for other analytes. Only bicarbonate did not meet the strictest (Royal College of Pathologists of Australasia Quality Assurance Program) performance criteria. This was due to poor precision rather than bias. There were no systematic differences between laboratory-derived (analytic) CV’s and the CV’s of our flown versus terrestrial sample pairs however CV’s from the sample pairs tended to be slightly higher than analytic CV’s. The overall concordance, based on clinical stratification (normal versus abnormal), was 97%. Length of flight had no impact on the results.

**Conclusions:**

Transportation of laboratory specimens via small UASs does not affect the accuracy of routine chemistry, hematology, and coagulation tests results from selfsame samples. However it results in slightly poorer precision for some analytes.

## Introduction

Unmanned Aerial Systems (UAS), colloquially known as drones, are aircraft without an on-board human pilot. On December 1^st^ 2013 Amazon.com introduced the world to the idea of civilian drones when its CEO unveiled Prime Air, a delivery drone, on live TV. However UAS are not new. They have been in use since the early 1900’s[1] but were primarily developed and flown by military organizations due to their enormous cost. Recent advances in technology have provided high quality sensors at low price-points, greatly expanding the availability and potential utility of UAS. Once of these potential new uses is the routine transport of small goods such as diagnostic clinical laboratory specimens.

Transport of biological specimens, whether by planes, trains, or cars, is ubiquitous in both high- and low-resourced environments[2–4]. The majority of specimens are obtained in physician offices or clinics that tend to have small laboratories with limited testing menus[5,6]. Thus samples must be transported to larger, more complex laboratories to provide the testing required for clinical care. To illustrate, there are approximately 244,000 laboratories in the United States[7]. In 2006, physician office and other small non-hospital clinical laboratories accounted for ~75% of the total number of laboratories[6,8], but they only accounted for 13% of the test volume. In addition 63% of their testing was in a point-of-care format which proffers a limited range of tests relative to core laboratory testing[6,8]. A 2011 survey of clinical laboratories in Kampala, Uganda showed the same pattern. Physician office laboratories (POL’s) accounted for 94% of clinical laboratories[9], but only accounted for 52% of the test volume[5] and > 80% of these POL’s performed only simple kit tests (point of care tests) or light microscope exams.

In addition to being a potentially new mode of transporting biological samples, UAS have unique advantages such as no traffic delays, low overhead costs, and the ability to go where there is no passable road. The impact of poor or difficult road access on healthcare is well documented in both high-[10] and low-resourced[11,12] countries. UAS are a potential way around this barrier, but are only useful if they do not adversely affect the test results of transported samples[2,13–16].

Our first challenge in addressing the impact of UAS transport on laboratory results was the high and expanding number of tests used in clinical care[17]. Fortunately, less than 0.5% (40/2000) of these tests account for 80% of the test volume. Thus we began by focusing, in these first experiments, on the impact of UAS transport on the 33 most common tests performed in hospital laboratories[17,18]. A second challenge was determining what quality criteria to use for evaluating any differences we might see. There is no single worldwide consensus on acceptable performance for laboratory tests. The most widely used performance criteria are intended for interpretation of External Quality Assessment reports. They are largely measures of accuracy, and vary by jurisdiction[19–22]. To account for these limitations, we evaluated our results in three ways. 1) We used four performance acceptability criteria including two from groups outside the United States[19–22]; 2) We examined changes in reference range-based clinical classification; and 3) We examined differences between laboratory-derived (analytic) CV’s and that from our paired samples.

To the best of our knowledge there has been no published research of the impact of UAS transportation on the stability of biological specimens or on the laboratory test results obtained from those specimens. Obtaining this data, which would be needed to determine the feasibility of UAS transportation of biological samples, is the objective of this study.

## Methods

### Study Design

All participants were orally consented using an identical script in English. Oral consent was used to guarantee anonymity for the volunteers. The samples were identified using a study ID and there was no key linking the participants to the samples or results. The consent procedure and the study were approved by the Johns Hopkins Medicine Human Subjects Institutional Review Board (Baltimore). 56 volunteers were recruited for the study: 36 females and 23 males. The mean age (SD) was 38.1 ± (11.6) years. Three paired samples (6 total) were obtained from each of the 56 adult volunteers: two 3.5 mL serum separator tubes, two 3 mL Potassium EDTA whole blood tubes, and two 2.7 mL citrated plasma tubes (BD Vacutainer). All six samples were collected in a single event using standard phlebotomy technique.

**Fig 1:**
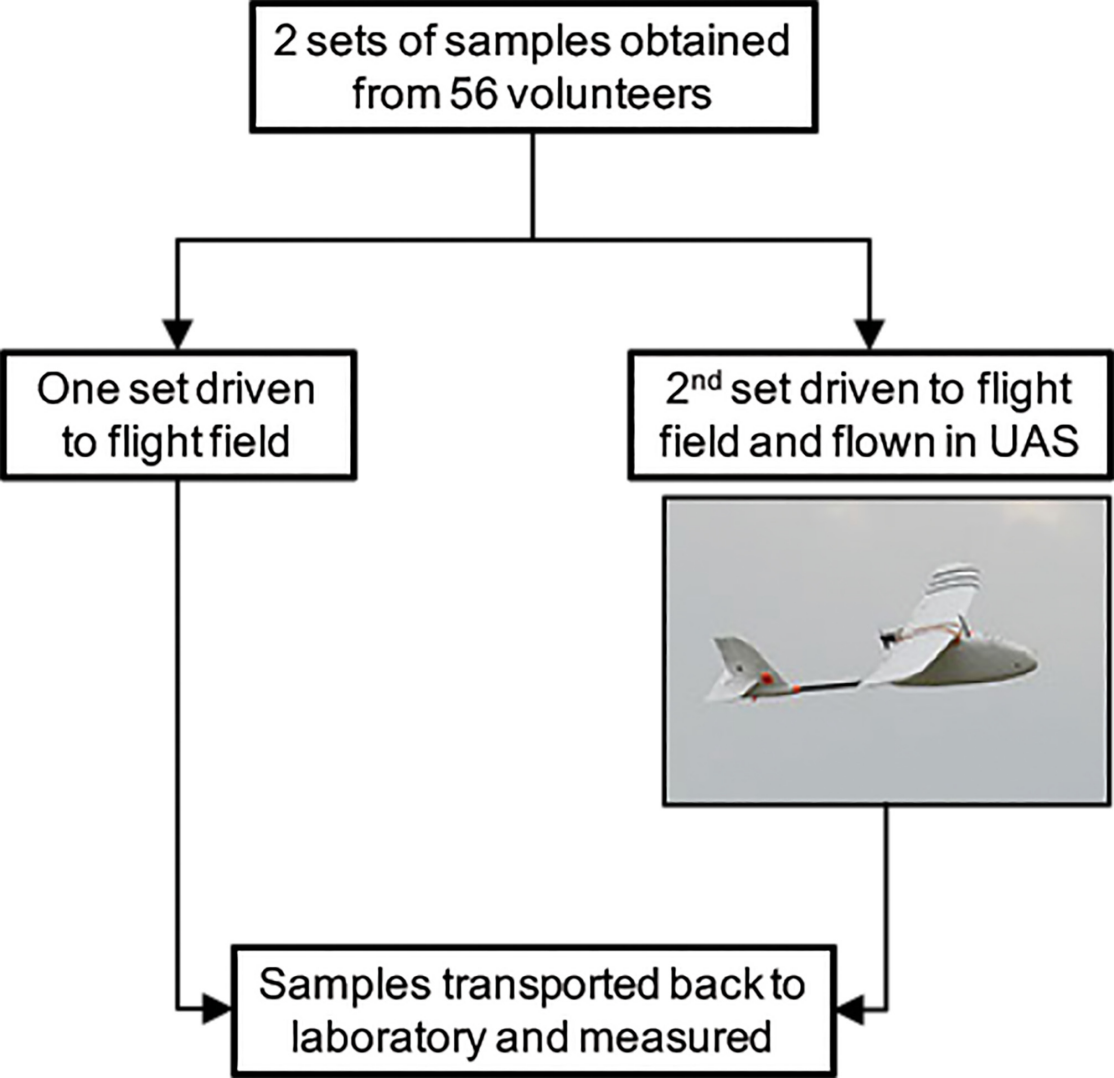
Schematic of the experiment.

One set of the paired tubes was driven to the flight site and flown in the UAS. The second sample set was driven to the flight site but not flown (Fig 1). Flight times were staggered, from a minimum of 6 to a maximum of 37.5 minutes. All samples were kept at ambient temperatures. The maximum temperatures in the transport vehicles and in the shade at the flight site on the two flight days were 76 and 79°F respectively.

**Fig 2:**
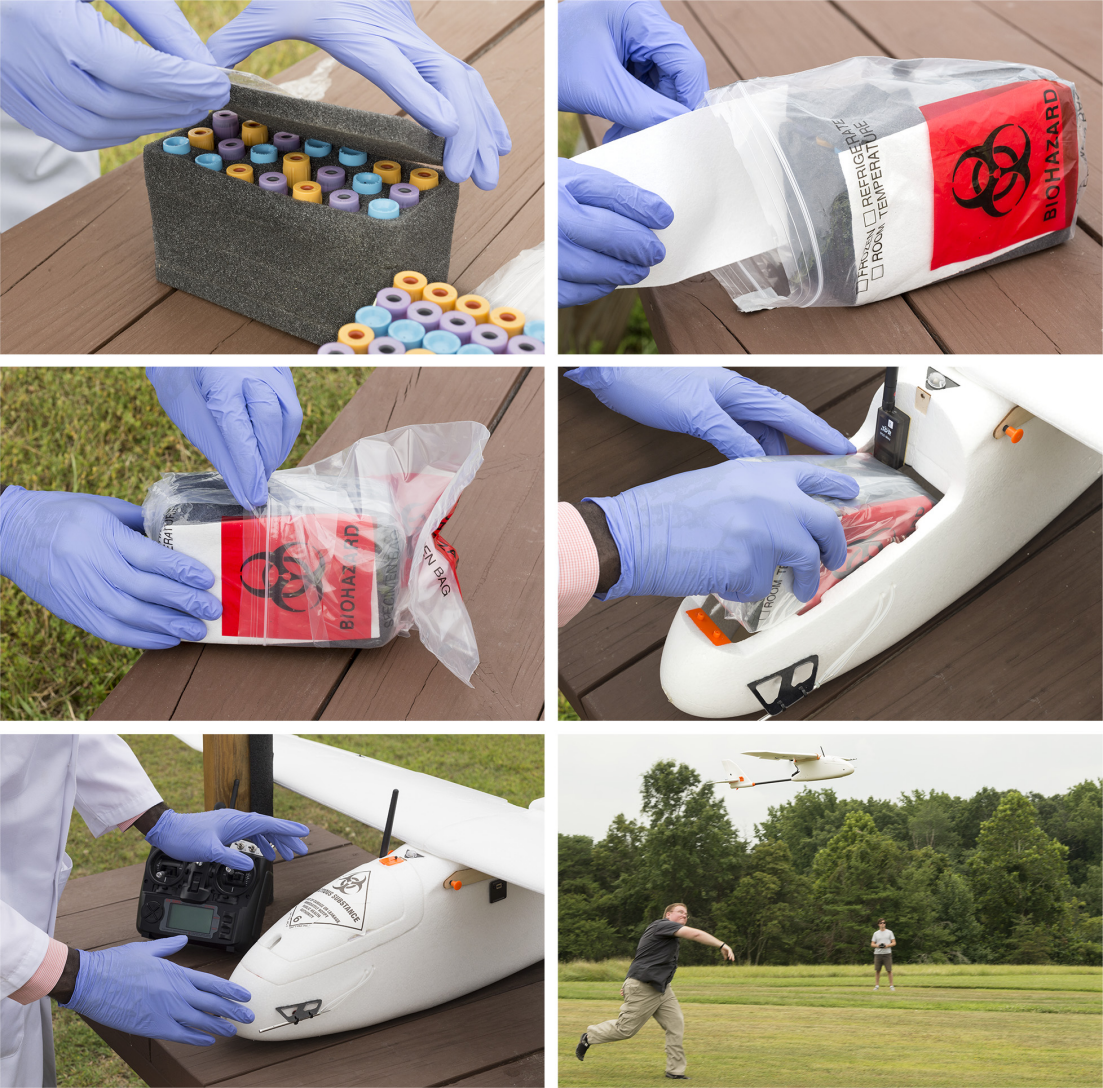
Figure showing the packing of the sample payload. 1. Custom-cut foam block. 2. Placement of sealed foam lock in the bio-hazard bags as well as absorbent material for potential sample containment. 3. Placement of first bio-hazard bag inside the second bio-hazard bag. 4. Placement of double-wrapped payload in the fuselage. 5. Covered, secured, and labeled fuselage. 6. Launch with hand toss.

For flight, the samples were packed in a sample payload module which served to control the in-flight environment as well as to contain the samples in the unlikely event of a leak or breakage (Fig 2). The flights were conducted in compliance with Advisory Circular (AC) 91–57[23], Model Aircraft Operating Standards as well as the International Air Transport Association’s (IATA) Guidelines for the packaging of potentially infectious liquid biological materials (REF 6.1) (Fig 2) [24]. Briefly, each sample was enclosed by three layers of packaging and enough STP absorbent material (SAF-T-PAK, Hanover, MD 21076; http://www.saftpak.com/STPPack/) to absorb twice the full volume of all the samples in the payload. The primary receptacles were the original sample tubes, separated from each other by a custom-cut foam block. The secondary receptacles were two sealed biohazard bags wrapped in opposite orientations around all the Primary Receptacles. The tertiary receptacle was the rigid aircraft fuselage, made of impact absorbent EPS foam. Finally, the module carried an IATA label designating the contents as a class 6.2 infectious substance.

After flight operations were completed, all the samples (flown and stationary) were transported back to the Johns Hopkins Hospital Core laboratory. The time from the first drawn sample to the last result was less than 8 hours for all 336 samples in this experiment. The time from phlebotomy to arrival at the laboratory was uniform for sample sets from each individual but was not uniform across individuals. Serum and citrated plasma samples were centrifuged at 1900 × g for 7 minutes at 18.5°C and analyzed. Chemistry testing was performed on the Roche Hitachi c701 analyzer (Roche Diagnostics, Indianapolis, IN) and Hematology (CBC) testing performed on the Sysmex XN-9000 hematology analyzer (Sysmex America, Inc., Lincolnshire, IL). PT and aPTT measurements were made on BCS XP analyzers (Siemens Medical Solutions USA, Inc., Malvern, PA 19355).

### Flight Protocol

Samples were flown in a small fixed-wing aircraft (“Aero” from 3D Robotics, Berkeley, CA 94710; http://3drobotics.com) at an Altitude above Ground Level (AGL) of 100 meters. The aircraft was controlled using a conventional hobbyist 2.4GHz radio control link. A fixed-wing aircraft was selected over other aircraft types, such as helicopter or multi-rotor, because it has the best range capability for a given take-off weight, is least expensive, and is least mechanically complex.

The aircraft was launched with a hand toss, and flown up to the test altitude of 100 meters. It orbited the flight field, within the pilot’s visual range, for the duration of the test. At the end of the flight, it was brought down to land on the belly skid. Among other precautions, the test was conducted away from populated areas, the aircraft was under the control of a ground-based pilot, and the aircraft’s altitude was less than 100 meters.

### Statistical Analysis

Deming regression was used to compare flown with stationary results for Sodium, Potassium, Chloride, CO_2_ (bicarbonate), Blood Urea Nitrogen, Creatinine, Glucose, Calcium, Blood Urea Nitrogen/Creatinine, White Blood Cell (WBC), Red Blood Cell (RBC), Hemoglobin (Hb), Hematocrit (Hct), Mean corpuscular volume (MCV), Mean corpuscular Hemoglobin (MCH), Mean corpuscular Hemoglobin Concentration (MCHC), Red blood cell distribution width (RDW), Platelet count (Plt), Mean Platelet Volume (MPV), Lymphocyte %, Monocyte %, Neutrophil %, Eosinophil % (Eos%), Basophil % (Baso%), Lymphocytes (Lymph), Monocyte (Mono), Neutrophil (Neut), Eosinophil (Eos), Prothrombin Time (PT), and Activated Prothrombin Time (aPTT). Linear regression was used to compare results for Anion Gap, International Normalized Ratio (INR), and aPTT Ratio for flown versus stationary sample pairs. Linear regression was also used to investigate differences between flown and stationary samples pairs as a function of flight time. To determine if our results met clinical and regulatory quality criteria, we compared the 95% limits of agreement of our results to the intervals describing performance acceptability requirements for individual analytes [19–22]. To examine the repeatability of our results, we compared analytic CV’s based on repeat measurements of control material, to CV’s from our flown and terrestrial specimens. To determine the effect of UAS flight on the clinical classification of patients, we stratified patient groups according to their reference ranges; normal and abnormal, and compared agreement of the flown and stationary sample pairs (concordance). Analyse-it Software for Microsoft Excel Version 3.90.1 (Analyse-it Software, Ltd., Leeds, UK) and Excel (Microsoft, Redmond, WA) were used to do the analysis.

## Results

### Correlations

Tables 1, 2, and 3, show data describing the linear relationship between the flown and stationary sample chemistry, hematology, and coagulation results. The slopes of the regression equations were between 0.93 and 1.10 for all 33 tests and between 0.95 and 1.05 for 26 of the 33 tests. In addition, the intercept was close to zero: < 5% of the mean value for 31 of the 33 analytes, and <13% of the mean value for all analytes. Thus for these 26 tests, the results obtained from the flown and stationary sample pairs were within 5% of each other, and within 10% of each other for all 33 tests.

**Table 1:** Chemistry results from flown and stationary samples.

Analyte	Regression Equation (Flown = m*Terrestrial + b)	*R* ^*2*^	Laboratory Control		Flown and Stationary sample pairs	
			Mean	CV	Mean	CV*
**Sodium**	y = 1.02x–3	0.70	124.5 mmol/L	0.9	140.4 mmol/L	0.9
**Potassium**	y = 0.96x + 0.17	0.84	4.0 mmol/L	1.3	4.0 mmol/L	3.1
**Chloride**	y = 1.10x–9.9	0.81	97.2 mmol/L	0.9	99.4 mmol/L	1.0
**CO**_**2**_	y = 0.93x + 1.74	0.40	30.6 mmol/L	4.6	26.0 mmol/L	6.8
**Urea Nitrogen**	y = 1.0x–1.69	0.99	5.14 mmol/L	3.5	4.71 mmol/L	3.4
**Creatinine**	y = 0.98 + 0.02	0.95	79.6 μmol/L	4.0	70.7 μmol/L	4.9
**Glucose**	y = 1.0x–1.69	0.99	4.75 mmol/L	1.2	3.89 mmol/L	4.5
**Calcium**	y = 0.95x + 0.49	0.81	2.08 mmol/L	1.5	2.38 mmol/L	1.7
**Anion Gap**	y = 1.02x**	0.38			15.0 mmol/L	14.3
**SUN/Cr**	y = 0.99x + 0.034	0.97			17.5	6.0

Table 1. Summary of the chemistry results from flown and stationary samples; as well as analytic CV’s based on controls versus sample pairs.

*These are population CV’s.

**Simple linear regression

**Table 2:** Hematology results from flown and stationary samples.

Analyte	Regression Equation (Flown = m*Terrestrial + b)	*R* ^*2*^	Laboratory Control		Flown and Stationary sample pairs	
			Mean	CV	Mean	CV*
**WBC**	y = 0.99x + 0.05	0.99	7.0 × 10^9^/L	2.0	6.5 × 10^9^/L	3.3
**RBC**	y = 1.01x–0.04	0.99	4.4 × 10^12^/L	1.1	4.7 × 10^12^/L	1.3
**Hb**	y = 1.03x–0.36	0.99	7.9 mmol/L	1.0	8.3 mmol/L	1.0
**Hct**	y = 1.01x–0.34	0.95	0.37	1.6	0.42	2.2
**MCV**	y = 1.07x–6.18	0.97	84.4 fL	1.2	90.1 fL	1.6
**MCH**	y = 1.07x–2.25	0.99	29.1 pg/cell	1.3	28.7 pg/cell	1.2
**MCHC**	y = 0.99x + 0.31	0.74	345 g/L	1.7	31 9 g/L	2.3
**RDW**	y = 0.97x +0.39	0.99	0.152	0.9	0.140	0.9
**Plt**	y = 1.03x–7.18	0.96	245 × 10^9^/L	3.0	245 × 10^9^/L	4.6
**MPV**	y = 0.98x + 0.20	0.95	9.8 fL	1.9	11.0 fL	2.0
**Lymph%**	y = 0.99x +0.13	0.98	0.291	4.8	0.339	3.4
**Mono%**	y = 1.05x–0.26	0.92	0.138	9.9	0.075	8.4
**Neut%**	y = 1.00x–0.07	0.98	0.422	2.3	0.555	2.1
**Eos%**	y = 1.01x + 0.02	0.99	0.101	7.7	0.022	9.6
**Baso%**	y = 1.08x–0.05	0.64	0.048	2.7	0.007	24.1
**Lymph**	y = 0.98x + 0.02	0.98	2.0 × 10^9^/L	5.3	2.1 × 10^9^/L	4.5
**Mono**	y = 1.03x–0.01	0.95	1.0 × 10^9^/L	10.1	0.5 × 10^9^/L	9.0
**Neut**	y = 0.98x + 0.06	0.99	3.0 × 10^9^/L	3.0	3.6 × 10^9^/L	3.5
**Eos**	y = 1.07x–0.01	0.99	0.7 × 10^9^/L	7.9	0.2 × 10^9^/L	13.9

Table 2. Summary of the hematology results from flown and stationary samples; as well as analytic CV’s based on controls versus sample pairs.

*These are population CV’s.

**Table 3:** Coagulation results from flown and stationary samples.

Analyte	Regression Equation(Flown = m*Terrestrial + b)	*R* ^*2*^	Laboratory Control		Flown and Stationary sample pairs	
			Mean	CV	Mean	CV*
**PT**	y = 1.06x + 0.89	0.76	11.5 sec	1.4	10.3 sec	2.4
**INR**	y = 1.00x**	0.26	1.2	1.8	1.0	4.4
**aPTT**	y = 0.95x +1.15	0.74	27.7 sec	2.5	24.7 sec	3.5
**aPTT ratio**	y = 0.99x**	0.43	1.04	4.4	0.9	5.8

Table 3. Summary of the coagulation results from flown and stationary samples; as well as analytic CV’s based on versus sample pairs.

*These are population CV’s.

**Simple linear regression

21 of the 33 tests had coefficients of determinations (r^2^) above 0.9 and six of the 33 tests had coefficients of determinations had (r^2^) less than 0.7 between the results from the flown and stationary sample pairs.

Tables 1, 2, and 3, also show the between-run Coefficients of Variation (CV) of a normal control material for each of the 33 measured analytes compared to the population CV of stationary versus flown sample pairs.[25] Eight chemistry analytes were directly measured tests with controls. For six of these eight analytes (Table 1), the analytic CV was lower than the population CV, however the differences were small. The absolute differences between the analytic CV and the population CV ranged from 0.0 to 3.3 across the eight chemistry analytes.

19 hematology analytes were directly measured or calculated tests with controls. For 11 of these 19 analytes (Table 2), the population CV was lower than the analytic CV. The absolute differences between the analytic CV and the population CV ranged from 0.0 to 1.6 for 17 of these 19 analytes. The other two analytes, percent Basophil and percent Eosinophil, had differences of 21.4 and 6.0 respectively. The difference between the means of the laboratory controls and the population means were large: 9-fold (4.8/0.7) and 5-fold (0.71/0.15) respectively.

Four coagulation analytes were tests with controls. For all of these four analytes (Table 3), the population CV was higher than the analytic CV. The magnitude of difference between the analytic CV and the population CV ranged from 0.91 to 2.64 across the four analytes.

### Bland-Altman Comparisons

**Fig 3:**
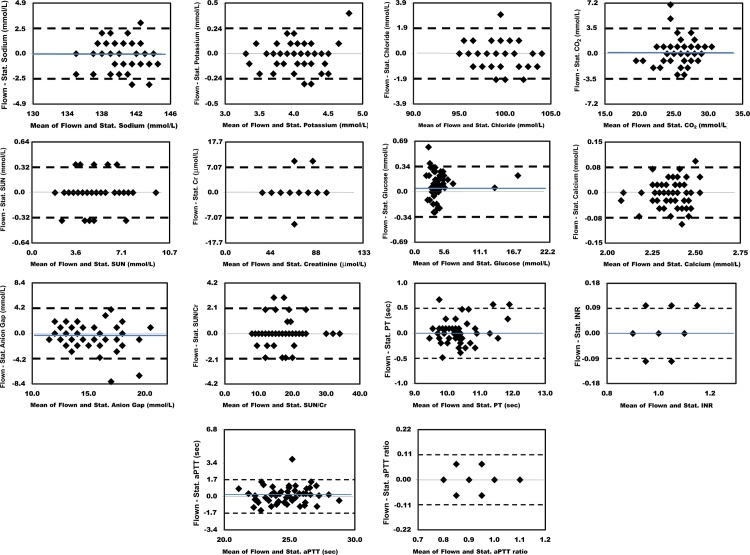
Bland Altman plots showing absolute differences in results for 56 flown versus stationary sample pairs. The dashed lines delineate the 95% limits of agreement. The blue lines show the mean difference for analytes where this was > 0.2% of the mean values for each analyte. 1, Sodium. 2, Potassium. 3, Chloride. 4, Carbon Dioxide. 5, Urea Nitrogen. 6, Creatinine. 7, Glucose. 8, Calcium. 9, Anion Gap. 10, SUN/Cr. 11, PT. 12, INR. 13, aPTT. 14, aPTT ratio.

**Fig 4:**
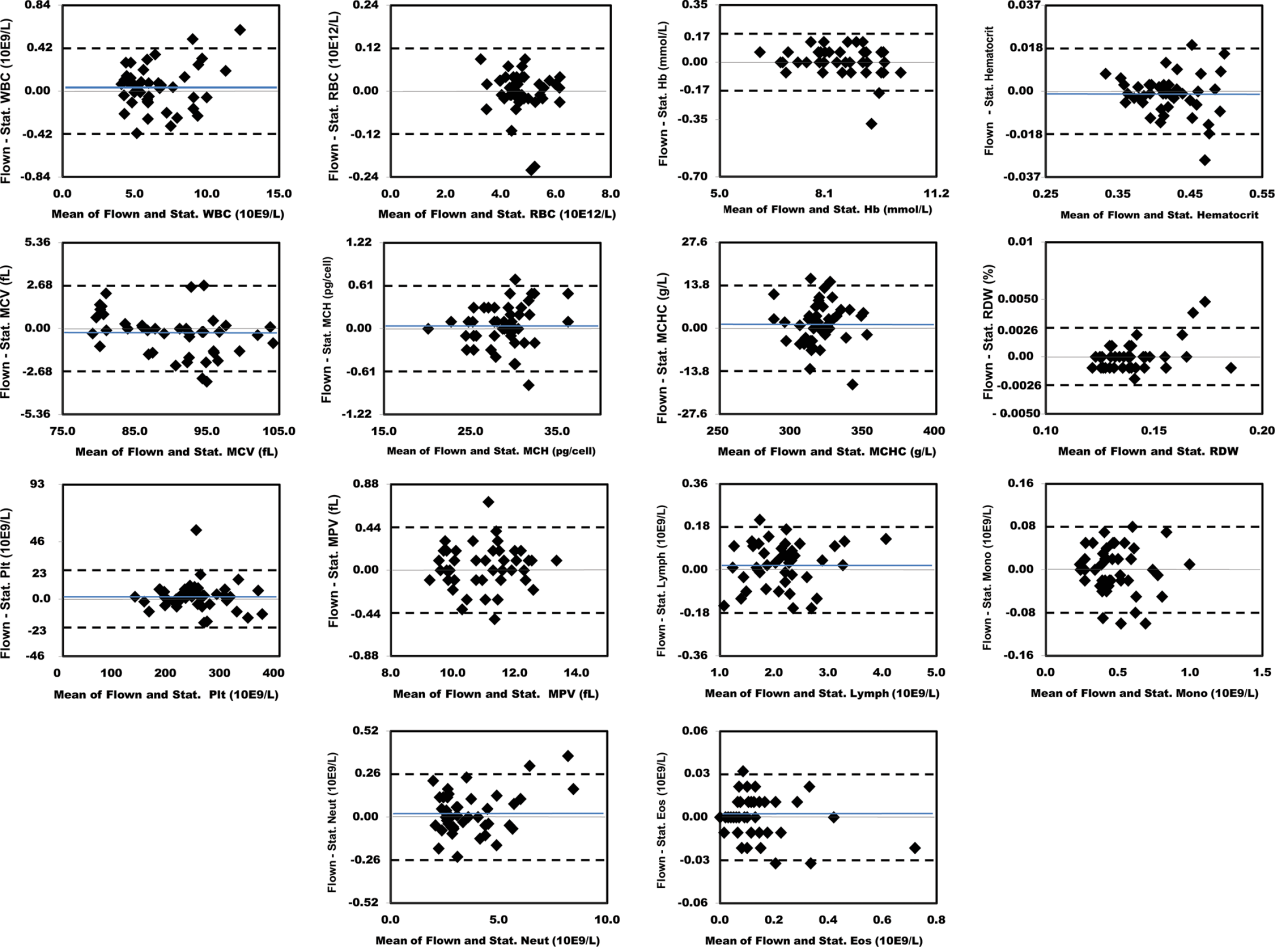
Bland Altman plots showing absolute differences in results for 56 flown versus stationary sample pairs. The dashed lines delineate the 95% limits of agreement. The blue lines show the mean difference for analytes where this was > 0.2% of the mean values for each analyte. 1, WBC. 2, RBC. 3, Hb. 4, Hct. 5, MCV. 6, MCH.7, MCHC. 8, RDW. 9, Platelet count. 10, MPV. 11, Lymphocytes. 12, Monocytes. 13, Neutrophils. 14, Eosinophils.

**Fig 5:**
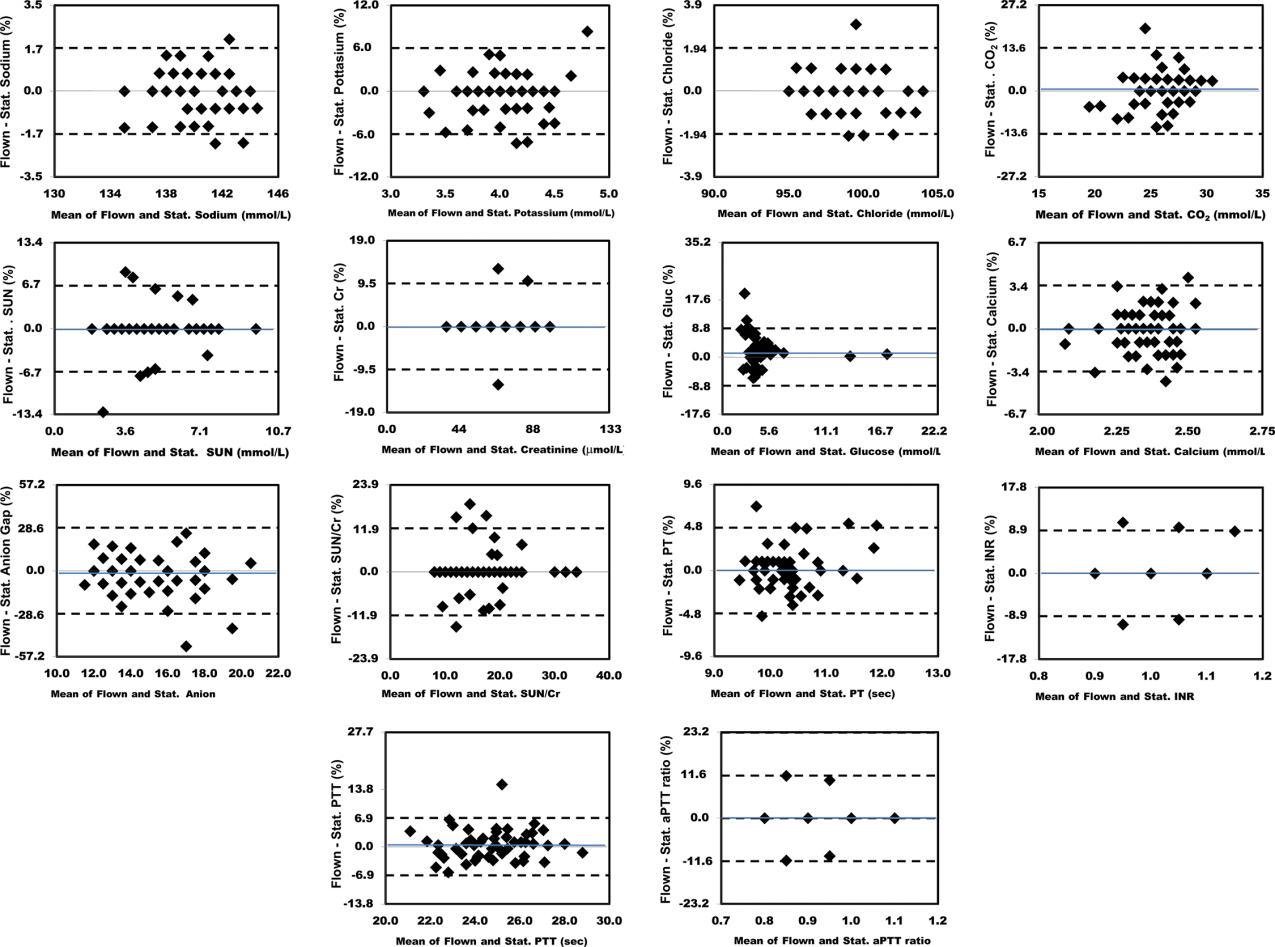
Bland Altman plots showing percent differences in results for 56 flown versus stationary sample pairs. The dashed lines delineate the 95% limits of agreement. The blue lines show the mean difference for analytes where this was > 0.2%. 1, Sodium. 2, Potassium. 3, Chloride. 4, Carbon Dioxide. 5, Urea Nitrogen. 6, Creatinine. 7, Glucose. 8, Calcium. 9, Anion Gap. 10, SUN/Cr. 11, PT. 12, INR. 13, aPTT. 14, aPTT ratio.

**Fig 6:**
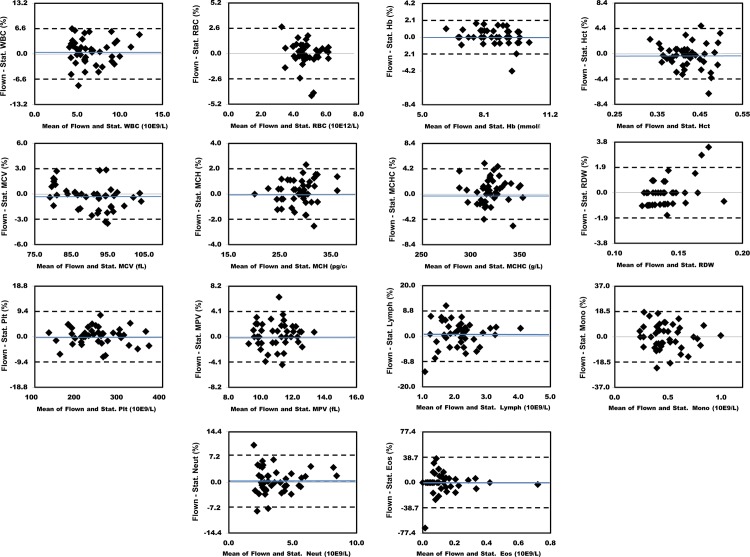
Bland Altman plots showing percent differences in results for 56 flown versus stationary sample pairs. The dashed lines delineate the 95% limits of agreement. The blue lines show the mean difference for analytes where this was > 0.2%. 1, WBC. 2, RBC. 3, Hb. 4, Hct. 5, MCV. 6, MCH.7, MCHC. 8, RDW. 9, Platelet count. 10, MPV. 11, Lymphocytes. 12, Monocytes. 13, Neutrophils. 14, Eosinophils.

Figs 3 and 4 show the absolute differences in the results obtained between individual flown and stationary sample pairs. The dashed lines delineate the 95% limits of agreement. The blue lines show the mean difference for analytes where this was > 0.2% of the mean value. Figs 5 and 6 show the percent differences in the results obtained between individual flown and stationary sample pairs. The dashed lines delineate the 95% limits of agreement. Only Glucose had a mean difference > 1.0%. Its mean difference was 3.2%. The blue lines show the mean difference for analytes where this was > 0.2% of the mean value.

Two of the eight chemistry analytes that correspond to measured tests, (CO_2_ and Glucose) had a 95% limit of agreement greater than 10%. Two of the 14 hematology analytes that correspond to measured or calculated, non-transformed tests, (Eosinophil and Basophil) had a 95% limit of agreement greater than 10%. Of note, these two analytes are also the two with the lowest mean levels. Three of the four coagulation analytes that correspond to measured or calculated non-transformed tests, (aPTT ratio) had a 95% limit of agreement greater than 10%.

### Allowable Performance Limits

With the exception of CO_2_ (bicarbonate), the 95% intervals for sample pair differences seen in this study were less than the performance criteria we used for comparisons[19–22]. The strictest bicarbonate ‘Allowable Limits’ criterion, the Royal College of Pathologists of Australasia Quality Assurance Programs’, was 10%. The 95% interval for bicarbonate in our study was 13.6%.

### Sample Concordance

Table 4 shows the concordance of the results from the stationary versus flown sample pairs using reference-range defined normal and abnormal cohorts. The overall concordance between results from the stationary and flown sample sets was 97% overall, 98.4% for normal results and 86% for abnormal samples.

**Table 4:** Concordance Between flown and stationary results.

		Flown Chemistry		Group concordance	Number
Stationary		Normal	Abnormal		
**Chemistry**	Normal	431	12	97%	443
	Abnormal	8	44	85%	52
	Total				
		Normal	Abnormal		
**Hematology**	Normal	956	12	99%	968
	Abnormal	15	126	89%	141
	Total	971	138		
		Normal	Abnormal		
**Coagulation**	Normal	204	2	99%	206
	Abnormal	6	8	57%	14
	Total	210	10		

### Flight Time

The length of flight had no measurable impact on the differences between results from flown and stationary samples. S1 Fig illustrates this finding for Sodium. The pattern was the same for all analytes.

## Discussion

This report examines the effect of small UAS transport on the 33 most common chemistry, hematology, and coagulation clinical laboratory tests. The results from flown versus stationary sample pairs were compared using several statistical approaches to determine the presence and magnitude of any differences between them. In particular we were examined three kinds of errors; systematic bias, random errors, and changes in clinical classification. The laboratory results from stationary and flown sample pairs were similar and did not show any systematic biases. A few analytes; namely, Chloride, CO_2_, MCV, MCH, Basophil %, Eosinopil, and partial Thromboplastin time (aPTT) had a 95% limit of agreement >10%. However these analytes had low mean levels (Eosinophil and Basophil), high variability (CO_2_ and Monocytes), or were based on transformed data (aPTT ratio). Of the 21 directly measured (as opposed to calculated) analytes in this study, only CO_2_ (bicarbonate) failed to meet the ‘Allowable Limits’ criteria. However this was due to poorer precision rather than a systematic bias. It is likely that this reflects the intrinsic variability of the assay as well as prolonged time-to-analysis rather than changes in atmospheric carbon dioxide levels with altitude as these are small relative to the variances in our results[26,27].

Random errors resulted in slightly poorer precision in the experimental sample pairs compared to analytic CV’s. Presently we are unable to conclusively determine if this is due to UAS transport or protracted time from initial phlebotomy to analyte measurement. This is because the regulatory environment in which our experiment was performed, which limited UAS flights to specific unpopulated areas and resulted in protracted time-to-analysis, still exists. However it is clear that the impact on precision is small. The overall agreement when samples were stratified clinically (normal vs. abnormal) was 97%. The agreement for normal samples was 99%. The agreement for abnormal samples alone was significantly worse. However this reflects two limitations of our test cohort rather than a discrepancy in the clinical classification of abnormal samples due to UAS transport. There were very low numbers of abnormal samples, and the abnormal values tended to be just outside the reference range so variation in results between sample pairs would lead to a re-classification of the result even though the magnitude of the changes were small.

The coefficient of determination, r^*2*^ of 21 of 33 tests in this report met or exceeded an r^*2*^ *≥* 0.9. The other 12 tests had r^*2*^ values < 0.9, but these were for reasons that were unrelated to agreement between the two result sets. r^*2*^ constant is also affected by a low mean value of a cohort, a narrow range of values (highest–lowest) in a cohort, or only a few possibilities within a cohort (e.g. a dichotomous variable). In our case, all 12 of these tests with r^*2*^ <0.9, had either low mean normal values, narrow normal range, or relatively few possible values within that range (S1 Table). For example, the tests INR, and RDW, both have a (reference Range / Pop. Mean) of 0.2. However, there are only three possible values for an INR in the normal range while there are 30 possible values for RDW in its normal range. Thus the r^*2*^ for INR is 0.263 while that for RDW is 0.993.

At the inception of this work, there was no precedent for packaging samples for UAS transport. To address this, we considered environmental variables that might be relevant for this mode of transportation including temperature, atmospheric pressure, and acceleration. Temperature change with altitude, or Adiabatic Lapse Rate, is small (0.6°C/100m) at elevations from 0–12,000m[28]. Atmospheric pressure change with altitude is also small for small changes in altitude, about 1.2 kPa (0.012 atm) for every 100 meters[29–31]. Our test flights called for changes in altitude that were less than 100 m, therefore we reasoned that no specific measures would be needed to stabilize temperature or pressure when ambient conditions were not extreme. However, we anticipated that acceleration might be a significant environmental factor because the UAS was launched by a hand toss, and landed by sliding to a stop on its belly (Fig 1 and https://vimeo.com/123492106). To mitigate these effects, we packed the sample vials individually in custom-cut soft foam (Fig 1), sealed this in two flexible biohazard bags (zip-loc) with absorbent material, and placed this package inside the fuselage, which is constructed of impact absorbing EPS foam (e.g. Styrofaom). Under the conditions of our experiment, there was no impact of the flight on hemolysis rates. This was determined by comparing hemolysis indices of the flown and stationary sample sets as measured on the Roche Hitachi c701 analyzer. It is likely that the custom-cut soft foam scaffold used to hold the tubes helped to stabilize them in transit. Thus any adoption of UAS transport of clinical diagnostic specimens will need to follow similar practices.

This study’s most significant limitation is that the volunteers were mostly healthy individuals and so their results were in the relatively narrow normal range, rather than spread across the full assay range (low to high) for each test. Thus we do not know the impact of UAS transport on results that are outside of the normal reference range. Subsequent experiments will be required to flesh out changes across the full reporting range (low to high) of each test-type. Nevertheless this paper is an important first step in determining if laboratory tests for the most common analytes used in healthcare are reliable when those samples are transported by UAS.

## Conclusions

Our findings demonstrate that, for the 33 test-types in this study, laboratory results from UAS-transported samples agree with those transported terrestrially: there were no systematic differences in results from flown versus terrestrial specimens. However, there was slightly worse precision in the flown samples. Full adoption of UAS transport of diagnostic specimens will require similar studies for other types of laboratory tests, specimens, and environmental conditions.

## Supporting Information

S1 Data Database of the raw data generated in this experiment. (XLSX)

S1 Fig Difference plot showing percent differences in Sodium results for 56 flown versus stationary sample pairs. The x-axis is the flight time. The dashed lines delineate the 95% limits of agreement. The blue lines show the trendline of the difference versus flight times
(EPS)

S1 Table Showing the size of normal range, the mean analyte level and the R^2^ for all analytes. (DOCX)
