# Supplementary material for: Can Unmanned Aerial Systems (Drones) Be Used for the Routine Transport of Chemistry, Hematology, and Coagulation Laboratory Specimens?
Source: PLoS One. 2015 Jul 29;10(7):e0134020. doi: 10.1371/journal.pone.0134020 (PMC4519103; doi:10.1371/journal.pone.0134020)
Supplement: S1 Table — (DOCX) [file pone.0134020.s003.docx]

| **Analyte** | **Size of NL range** | **Unflown Mean** | **NL Range / Pop. Mean** | ***R^2^*** |
| --- | --- | --- | --- | --- |
| **Sodium** | 13 | 140.4 mmol/L | 0.09 | 0.70 |
| **Potassium** | 1.6 | 4.0 mmol/L | 0.40 | 0.84 |
| **Chloride** | 13 | 99.4 mmol/L | 0.13 | 0.81 |
| **Carbon Dioxide** | 10 | 26 mmol/L | 0.38 | 0.40 |
| **Urea Nitrogen** | 15 | 4.71 mmol/L | 1.14 | 0.99 |
| **Creatinine** | 0.8 | 70.7 mmol/L | 1.00 | 0.95 |
| **Glucose** | 40 | 3.89 mmol/L | 0.57 | 0.99 |
| **Calcium** | 2.1 | 2.4 mmol/L | 0.22 | 0.81 |
| **Anion Gap** | 9 | 15 mmol/L | 0.60 | 0.38 |
| **SUN/Cr** |  | 17.5 | 0.00 | 0.97 |
| **WBC** | 6.5 | 6.5 × 10^9^/L | 1.00 | 0.99 |
| **RBC** | 1.3 | 4.7 10^12^/L | 0.28 | 0.99 |
| **Hb** | 3.6 | 8.32 mmol/L | 0.27 | 0.99 |
| **Hct** | 11 | 0.42 | 0.26 | 0.95 |
| **MCV** | 20 | 90.1 fL | 0.22 | 0.97 |
| **MCH** | 8 | 28.7 pg/cell | 0.28 | 0.99 |
| **MCHC** | 6 | 31.9 g/L | 0.19 | 0.74 |
| **RDW** | 3 | 0.14 | 0.21 | 0.99 |
| **Plt** | 200 | 245 × 10^9^/L | 0.82 | 0.96 |
| **MPV** | 3.5 | 11 fL | 0.32 | 0.95 |
| **Lymph%** | 20 | 0.34 | 0.59 | 0.98 |
| **Mono%** | 9 | 0.075 | 1.20 | 0.92 |
| **Neut%** | 53 | 0.56 | 0.96 | 0.98 |
| **Immature Gran%** | 1 | 0.0022 | 4.55 | 0.58 |
| **Eos%** | 3 | 0.022 | 1.36 | 0.99 |
| **Baso%** | 2 | 0.0071 | 2.82 | 0.64 |
| **Lymph** | 3.7 | 2.12 × 10^9^/L | 1.75 | 0.98 |
| **Mono** | 1.1 | 0.48 × 10^9^/L | 2.29 | 0.95 |
| **Neut** | 6.3 | 3.63 × 10^9^/L | 1.74 | 0.99 |
| **Immature Gran** | 0.05 | 0.02 × 10^9^/L | 2.50 | 0.52 |
| **Eos** | 0.18 | 0.15 × 10^9^/L | 1.20 | 0.99 |
| **PT** | 2.2 | 10.32 sec | 0.21 | 0.76 |
| **INR** | 0.2 | 1.01 | 0.20 | 0.26 |
| **aPTT** | 7.7 | 24.71 sec | 0.31 | 0.74 |
| **aPTT ratio** |  | 0.93 | 0.00 | 0.43 |

Supplementary Table 1: Showing the size of normal range, the mean analyte level and the R^2^ for all analytes.
